# Plasma neurofilament light and phosphorylated tau 181 as biomarkers of Alzheimer’s disease pathology and clinical disease progression

**DOI:** 10.1186/s13195-021-00805-8

**Published:** 2021-03-25

**Authors:** Christopher Clark, Piotr Lewczuk, Johannes Kornhuber, Jonas Richiardi, Bénédicte Maréchal, Thomas K. Karikari, Kaj Blennow, Henrik Zetterberg, Julius Popp

**Affiliations:** 1 Institute for Regenerative Medicine, University of Zürich, Zürich, Switzerland; 2 Department of Psychiatry and Psychotherapy, Universitätsklinikum Erlangen, and Friedrich - Alexander Universität Erlangen-Nürnberg, Erlangen, Germany; 3 Department of Neurodegeneration Diagnostics, Medical University of Białystok, Białystok, Poland; 4 Department of Radiology, Lausanne University Hospital and University of Lausanne, Lausanne, Switzerland; 5 Advanced Clinical Imaging Technology group, Siemens Healthcare AG, Lausanne, Switzerland; 6 LTS5, École Polytechnique FÉdÉrale de Lausanne (EPFL), Lausanne, Switzerland; 7 Department of Psychiatry and Neurochemistry, Institute of Neuroscience & Physiology, the Sahlgrenska Academy at the University of Gothenburg, Mölndal, Sweden; 8 Clinical Neurochemistry Laboratory, Sahlgrenska University Hospital, Mölndal, Sweden; 9 Department of Neurodegenerative Disease, UCL Institute of Neurology, London, UK; 10 UK Dementia Research Institute at UCL, London, UK; 11 Old age Psychiatry, Department of Psychiatry, University Hospital of Lausanne, Lausanne, Switzerland; 12 Department of Geriatric Psychiatry, University Hospital of Psychiatry Zürich and University of Zürich, Zürich, Switzerland

**Keywords:** Biomarkers, Plasma, Alzheimer’s, Neurofilament light, p-tau181

## Abstract

**Background:**

To assess the performance of plasma neurofilament light (NfL) and phosphorylated tau 181 (p-tau181) to inform about cerebral Alzheimer’s disease (AD) pathology and predict clinical progression in a memory clinic setting.

**Methods:**

Plasma NfL and p-tau181, along with established cerebrospinal fluid (CSF) biomarkers of AD pathology, were measured in participants with normal cognition (CN) and memory clinic patients with cognitive impairment (mild cognitive impairment and dementia, CI). Clinical and neuropsychological assessments were performed at inclusion and follow-up visits at 18 and 36 months. Multivariate analysis assessed associations of plasma NfL and p-tau181 levels with AD, single CSF biomarkers, hippocampal volume, and clinical measures of disease progression.

**Results:**

Plasma NfL levels were higher in CN participants with an AD CSF profile (defined by a CSF p-tau181/Aβ_1–42_ > 0.0779) as compared with CN non-AD, while p-tau181 plasma levels were higher in CI patients with AD. Plasma NfL levels correlated with CSF tau and p-tau181 in CN, and with CSF tau in CI patients. Plasma p-tau181 correlated with CSF p-tau181 in CN and with CSF tau, p-tau181, Aβ_1–42_, and Aβ_1–42_/Aβ_1–40_ in CI participants. Compared with a reference model, adding plasma p-tau181 improved the prediction of AD in CI patients while adding NfL did not. Adding p-tau181, but not NfL levels, to a reference model improved prediction of cognitive decline in CI participants.

**Conclusion:**

Plasma NfL indicates neurodegeneration while plasma p-tau181 levels can serve as a biomarker of cerebral AD pathology and cognitive decline. Their predictive performance depends on the presence of cognitive impairment.

## Background

In vivo detection of the cerebral pathophysiological processes of Alzheimer’s disease (AD) is key to accurate diagnosis and appropriate care. Cerebrospinal fluid (CSF) and positron tomography biomarkers of amyloid and tau accurately detect AD, but are of limited use in clinical practice due to the associated costs, invasiveness, or non-availability of the tools needed [1]. Non-invasive blood-based biomarkers could provide an attractive alternative, allowing to identify patients that may benefit from further, more invasive and/or costly diagnosis, or for recruitment and monitoring of participants in clinical trials [2].

Neurofilament light (NfL) protein and tau phosphorylated at threonine 181 (p-tau181) are promising candidates for blood-based biomarkers of AD. NfL blood level has been proposed as a biomarker for axonal damage and neuronal injury [2] and has been found to be increased in clinically diagnosed AD compared with healthy controls [3–6]. It also has been associated with cognitive decline in participants with normal cognition [7], and neurodegeneration across neurodegenerative diseases [8]. Plasma p-tau181 has been recently reported to be increased in both clinically diagnosed [9] and biomarker confirmed AD dementia [10], and to correlate with CSF tau levels and amyloid PET measurements [11, 12]. Furthermore, it may predict disease progression and cognitive decline in cognitively unimpaired participants and MCI patients [13].

Here, our aim was to test the ability of plasma NfL and plasma p-tau181 levels, or the combination thereof, to serve as blood-based biomarkers for the diagnosis of cerebral AD pathology and the prediction of clinical disease progression.

## Materials and methods

### Study population

Two hundred and twenty-one individuals aged 49 to 88 years were recruited at the memory clinic of the Department of Psychiatry and the Department of Clinical Neurosciences at the University Hospital of Lausanne, Switzerland into an AD biomarker discovery study cohort between 2014 and 2018. Participants were recruited among memory clinic patients and through advertising and word-of-mouth for healthy participants. All participants underwent a comprehensive clinical evaluation and neuropsychological assessment as previously described [14]. Briefly, a comprehensive test battery along with standard questionnaires were used to determine the Clinical Dementia Rating (CDR [15]), CDR sum of boxes (CDRSoB), Mini-Mental State (MMSE), and to verify subgroup inclusion criteria. The cognitive impairment group (CI) included patients with the clinical diagnoses of mild cognitive impairment (MCI [16], *n* = 56) or dementia (*n* = 71) and a CDR score ≥ 0.5 [14]. Patients with major psychiatric or neurological disorders, substance abuse, or severe or unstable physical illness that could affect cognition were excluded. Cognitively normal participants (CN) were free of relevant acute psychiatric or neurologic affection, had neither current cognitive impairment nor a history of it, and had a CDR = 0. MRI and CT scans were performed in all participants and used to exclude individuals with major cerebral pathologies possibly interfering with the cognitive performance. Clinical and neuropsychological evaluations were repeated after roughly 18 and 36 months, during follow-up visits using the same study protocol.

### Blood and cerebrospinal fluid collection

Venous and lumbar punctures were performed after an overnight fast. Ten to twelve milliliters of CSF was collected for analysis, centrifuged at 4 °C, immediately aliquoted, and frozen at − 80 °C until assayed, as previously described [17].

### CSF AD biomarkers, albumin quotient, and Apolipoprotein E genotype

CSF β-amyloid 1–42 peptide (Aβ_1–42_), total-tau (tau), and tau phosphorylated at threonine 181 (p-tau181) concentrations were measured using commercially available ELISA kits (Fujirebio Europe, Gent, Belgium). Additionally, the concentrations of Aβ_1–42_ and Aβ_1–40_ were measured with immunoassays from IBL International (Hamburg, Germany) according to the manufacturer’s protocols. The albumin CSF/serum quotient (QAlb) as a marker of blood-CSF barrier function along with the apolipoprotein E (*APOE*) genotype were determined as previously described [18].

### Plasma biomarkers

NfL concentrations were measured using the NF-light™ kit on a Single molecule array (Simoa) HD-X Analyzer (Quanterix, Billerica, MA, USA), following the recommendations by the manufacturer. Plasma p-tau181 levels were measured using an in house Simoa assay as previously described [10]. Briefly, an AT270 mouse monoclonal antibody (MN1050; Invitrogen, Waltham, MA, USA) was coupled to paramagnetic beads (103,207; Quanterix) and used for capture. As the detector, we used the anti-tau mouse monoclonal antibody Tau12 (806,502; BioLegend, San Diego, CA, USA), conjugated to biotin (A3959; Thermo Fisher Scientific, Waltham, MA, USA), while GSK-3β phosphorylated full-length recombinant tau441 (TO8–50FN; SignalChem, Vancouver, BC, Canada) was used as calibrator. Fluorescent signals were converted to average enzyme per bead numbers as described [19], and specimen concentrations extrapolated from four-parametric logistic curves generated with known calibrator concentrations.

### Hippocampal volume measurements

All participants underwent a magnetic resonance imaging scan at inclusion on a 3 T MRI system (MAGNETOM Prismafit, Siemens Healthcare, Erlangen, Germany) with a 32-channel head coil. Acquisitions followed the ADNI2 MRI protocol (http://adni.loni.usc.edu/methods/documents/). Images were segmented with the MorphoBox prototype [20], and both overall image [21] and segmentation quality were automatically assessed [20]. Here we used regional volumetric data normalized by total intracranial volume (defined as the sum of gray matter, white matter and CSF) to determine relative hippocampal volume.

### Data and statistical analysis

Before analysis, outliers for CSF and plasma biomarker levels (i.e., data points that exceeded the cut-off value of mean ± 3 × SD; 12 out of 205 for plasma NfL and 8 out 201 for plasma p-tau181 levels were concerned by this change, accounting for less than 3% of all data points) were replaced by the cut-off value to minimize quantification errors. All participants within the CN and CI subgroups were further classified as AD or non-AD according to the presence or absence of an AD CSF profile. An AD CSF profile was defined by a CSF p-tau181/Aβ_1–42_ ratio > 0.0779. This cut-off value was internally established as previously described [17]. Briefly, this value was determined using center data obtained from one hundred and twenty participants and was the value that optimized group separation based on the Youden index in this sample and was in line with previous publications [22]. Biomarker and cognitive change data were log-transformed prior to correlation and regression analyses to approach Gaussian distribution. Subgroups within the cohort were compared using Students’ two-tailed *t*-test for continuous variables and chi-square tests for categorical variables. Data are given as mean ± standard-deviation. Correlations between CSF AD biomarkers and plasma biomarkers were assessed using Spearman’s rho. Benjamini-Hochberg correction of *P*-value for multiple testing was applied for all analyses using a false-discovery rate of 0.1. Potential collinearity of the explanatory variables used in the regression modeling was tested with variance inflation factor (VIF). No variable entered in these models had VIF above 1.5; thus, the absence of multicollinearity was assumed. Statistical data analysis was performed with IBM SPSS Statistics software version 25.

### Statistical modeling

To assess the association of plasma NfL and plasma p-tau181 with the presence of AD pathology, we used logistic regression models with occurrence of AD as a dependent variable while entering both plasma markers as independent variables. We explored the effects of the following covariates: age, sex, years of education, and *APOE* ε4 status. Best predictive models were obtained using a backwards selection method where variables with a likelihood-ratio statistic probability > 0.1 were removed iteratively. A reference model for prediction of AD using only age, sex, years of education, and with or without *APOE* ε4 status was constructed using logistic regression in the CN and CI subgroups individually. We then added either plasma NfL levels or plasma p-tau181 levels, or both to this model. Predictive performance was assessed by computing a ROC curve and area under the curve (AUC) for these models and were compared using the DeLong method. Estimation of cut points for p-tau181 for the prediction of a CSF AD profile was done using R software (cutpointr package) and selecting the cutoff level that maximized the prediction accuracy of logistic regression models in CI participants.

Associations of plasma NfL and plasma p-tau181 with cognitive measurement changes were first assessed with linear regression models using CDRSoB or MMSE changes at the last follow-up visit as a dependent variable while entering both plasma markers as independent variables. We explored the effects of the following covariates: age, sex, years of education and *APOE ε*4 status, and baseline MMSE or CDRSoB scores, and time to follow-up. Best predictive models were obtained using a backwards selection method, where variables with a *F*-score statistic probability > 0.1 and the smallest correlation with the dependent variables were removed iteratively. In parallel, reference models for the prediction of clinical disease progression (CDRSoB change ≥1) or decline in global cognition (MMSE change ≥ − 2) using the above covariates were constructed in the CN and CI subgroups separately. We then added either plasma NfL levels or plasma p-tau181 levels, or both, to these models. Predictive performance was assessed using ROC and AUC values of models compared using the DeLong method as above.

Goodness-of-fit of logistic regression models was assessed using the Hosmer-Lemeshow test. None of the above models displayed a Hosmer-Lemeshow chi-squared value yielding a *P*-value < 0.05 and therefore none were rejected.

### Data availability

The datasets used and/or analyzed during the current study are available from the corresponding author upon reasonable request.

## Results

### Cohort description

Subject characteristics and cognitive assessments average by group comparisons, based on cognitive status at baseline together with AD CSF biomarkers, biochemical measures, and plasma NfL and p-tau181 levels, are shown in Table 1. Longitudinal clinical data from at least one FU visit after 36.68 ± 16.67 months in CN participants (*n* = 79) and 33.9 ± 16.07 months in CI (*n* = 94, *p*-value = 0.256) showed CDRSoB changes of 0.241 ± 0.76 and 2.88 ± 3.62 in CN and CI participants respectively (*p*-value < 0.001). For MMSE we observed a change of − 0.17 ± 1.16 in CN participants and of − 3.15 ± 5.60 in CI patients (*p*-value < 0.001). In CN participants both plasma NfL and p-tau181 levels positively correlated with age (spearman’s rho = 0.602 and 0.249, respectively), while in CI patients plasma NfL correlated with age and years of education (rho = 0.363 and − 0.243, respectively) and p-tau181 with *APOE* ε4 status (rho = 0.223). Neither plasma NfL nor plasma p-tau181 correlated with Qalb. Furthermore, Nfl levels correlated with CDR, CDRSoB, and MMSE scores (rho = 0.307, 0.44 and − 0.295, respectively) in CI patients.

**Table 1:** Characteristics of the study cohort

	CN (***n*** = 91)	CI (***n*** = 127)	***p***-value
Demographic and clinical characteristics			
Sex, female (%)	64.4	53.9	0.12
Age (years), mean ± SD	68.53 ± 7.31	74 ± 6.6	< 0.001
Years of education (years), mean ± SD	13.02 ± 2.54	12.26 ± 2.78	0.04
CDR, mean ± SD	0	0.59 ± 0.26	< 0.001
CDRSoB, mean ± SD	0.01 ± 0.07	2.12 ± 2.14	< 0.001
MMSE, mean ± SD	28.59 ± 1.25	25.29 ± 3.83	< 0.001
Biochemical measures			
*APOE* ε4, *n* (%)	18 (21.2)	50 (43.5)	0.001
QAlb, mean ± SD	5.45 ± 2.28	6.93 ± 3	0.002
CSF AD biomarkers			< 0.001
Aβ_1–42,_ pg/ml, mean ± SD	1030.62 ± 262.46	754.58 ± 287.32	< 0.001
Tau_,_ pg/ml, mean ± SD	301.09 ± 175.95	493.04 ± 308.79	< 0.001
p-tau181_,_ pg/ml, mean ± SD	57.74 ± 20.48	70.49 ± 29.77	< 0.001
Aβ_1–42_/Aβ_1–40_	0.07 ± 0.02	0.05 ± 0.02	< 0.001
MRI			
Hippocampal volume	0.0046 ± 46E-5	0.0041 ± 57E-5	< 0.001
Plasma biomarkers			
NfL, pg/ml, mean ± SD	17.61 ± 9.24	24.66 ± 11.66	< 0.001
p-tau181, pg/ml, mean ± SD	9.58 ± 7.09	14.78 ± 9.69	< 0.001

### Associations of plasma NfL and p-tau181 levels with AD

**Fig. 1:**
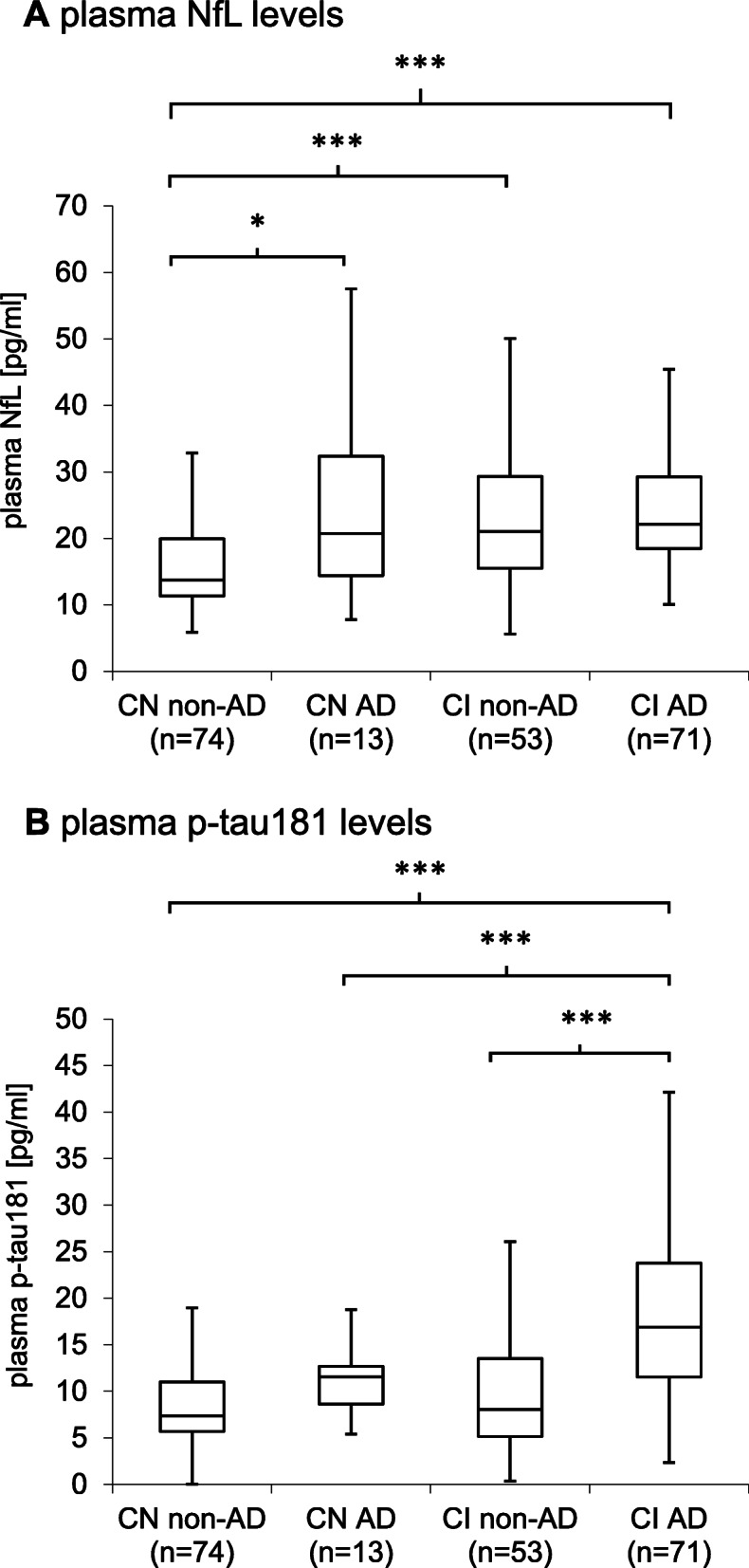
Plasma NfL and p-tau181 levels in the cohort. Boxplots of plasma NfL (**a**) and p-tau181 (**b**) concentrations in cognitively healthy participants (CN) and patients with cognitive impairment (CI). Both groups were further stratified according to AD CSF biomarker profile. Mean concentrations between pairs of groups were compared using *T*-tests. *, *p*-value < 0.05; ***, *p*-value < 0.001

**Fig. 2:**
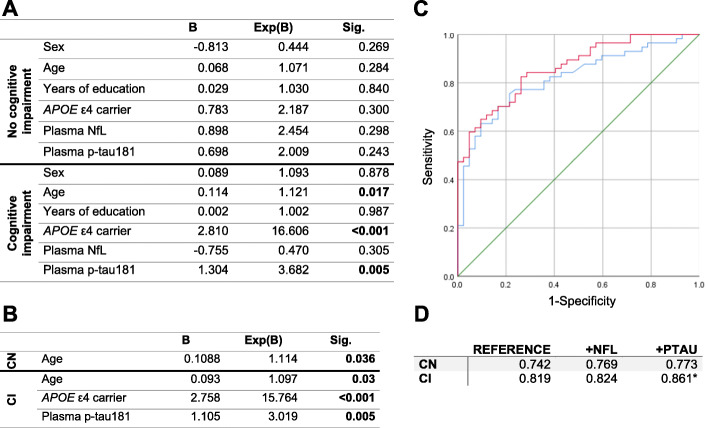
Models for the prediction of AD. **a**-**b** Binomial logistic regression models with the presence of AD pathology as dependent variable along with sex, age, years of education, *APOE* ε4 carrier status, plasma NfL and plasma p-tau181 levels, with no variable selection (**a**) or backwards variable selection (**b**). Coefficients (B) and odds-ratio (Exp(B)) are shown. **c** ROC curves from predictive models of the presence of AD in CI participants. The reference model (blue) and the reference model with p-tau-181 levels (red) are shown. The 0.5 AUC reference line is shown in green. ROC curves for CN participants for either models are not shown. **d** AUC of ROC curves obtained by the reference model (Reference) and after adding plasma NfL (+NFL) or p-tau181 levels (+PTAU) in both the CN and CI groups. *, *p*-value < 0.05

Plasma NfL levels were significantly higher in CN participants with cerebral AD pathology (as indicated by the presence of an AD CSF profile) and in both AD and non-AD CI patients when compared with CN non-AD participants (Fig. 1a). Plasma p-tau181 levels were significantly increased in CI participants with AD as compared with CI non-AD patients and both AD and non-AD CN participants (Fig. 1b). Hippocampal volume did not show significant differences between AD and non-AD subgroups in either CN or CI participants (data not shown). Within the CI group, no significant difference was observed for either Age, Sex or Years of education. When considering the following covariates in a regression model, age, sex, years of education, and *APOE* ε4 status only p-tau181 levels remained associated with AD pathology in CI participants (Fig. 2a). Applying backwards variable selection to both of these models identified age for CN participants and age, *APOE* ε4 status, and p-tau181 levels in CI participants as independent predictors of AD pathology (Fig. 2b). The addition of plasma p-tau181 levels to a reference model to predict the presence of AD pathology improved prediction accuracy in CI participants (Fig. 2c, d, *p*-value = 0.048; sensitivity: 0.8; specificity: 0.79, Delong’s ΔAUC = 0.042). Using a plasma p-tau181 cutoff at 9.68 pg/ml improved the prediction of AD (AUC = 0.869, *p*-value = 0.036; Delong’s ΔAUC = 0.051) in CI participants. Using this cutoff also improved the prediction of AD in the whole cohort (AUC = 0.861, *p*-value = 0.012; Delong’s ΔAUC = 0.049). Plasma NfL levels did not contribute to improving the prediction of AD (Fig. 2d), nor did adding both markers in combination (data not shown).

### Associations of plasma NfL and p-tau181 with CSF biomarkers of amyloid pathology, neuronal injury, and tau hyperphosphorylation

In the whole cohort, both plasma NfL and p-tau181 levels correlated with all assessed AD CSF biomarkers and with the hippocampal volume (data now shown). In both groups, plasma NfL and plasma p-tau181 levels were correlated (Table 2). In CN participants, plasma Nfl levels correlated with those of CSF tau and p-tau181, and plasma p-tau181 correlated with CSF p-tau181. In CI participants, plasma NfL levels correlated with CSF tau and hippocampal volume whereas plasma p-tau181 levels were correlated with all AD CSF biomarkers but not hippocampal volume (Table 2).

**Table 2:** Correlations (rho) of plasma NfL and plasma p-tau181 levels with other biomarkers

	Plasma NfL		Plasma p-tau181	
	rho	*p*-value	rho	*p*-value
CN participants				
CSF Aβ_1–42_	0.130	0.235	− 0.021	0.849
CSF Aβ_1–42/_Aβ_1–40_	−0.074	0.510	− 0.0173	0.128
CSF p-tau181	**0.242**	**0.026**	**0.326**	**0.003**
CSF tau	**0.26**	**0.016**	0.213	0.055
Plasma NfL			**0.317**	**< 0.001**
Plasma p-tau181	**0.317**	**< 0.001**		
Hippocampal volume	0.027	0.817	0.023	0.849
CI participants				
CSF Aβ_1–42_	−0.052	0.584	**0.415**	**< 0.001**
CSF Aβ_1–42_/Aβ_1–40_	−0.006	0.950	**− 0.328**	**< 0.001**
CSF p-tau181	0.148	0.116	**0.417**	**< 0.001**
CSF tau	**0.233**	**0.013**	**0.496**	**< 0.001**
Plasma NfL			**0.336**	**< 0.001**
Plasma p-tau181	**0.336**	**< 0.001**		
Hippocampal volume	**− 0.349**	**0.002**	−0.135	0.235

### Associations of plasma NfL and p-tau181 with disease severity progression and cognitive decline

**Fig. 3:**
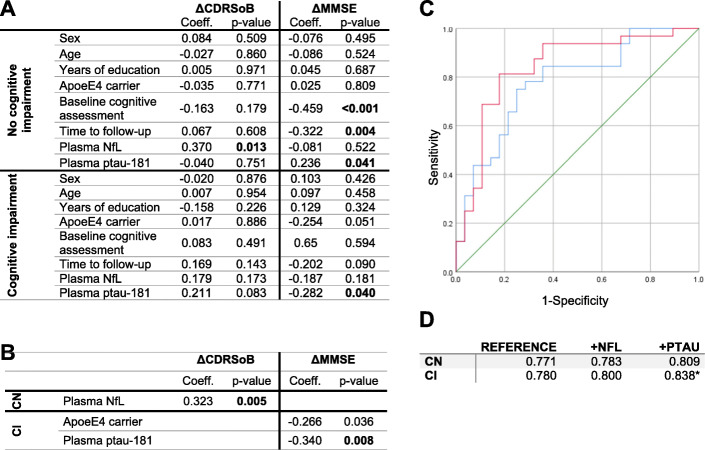
Models for the prediction of cognitive change. **a**-**b** Linear regression models with changes in cognition at last follow-up as a dependent variable along with sex, age, years of education, *APOE* ε4 carrier status, Baseline cognitive score, time to follow-up plasma NfL and plasma p-tau181 levels, with no variable selection (**a**) or backwards variable selection (**b**). Standardized coefficients, where the variances of dependent and independent variables are equal to 1 (Coeff.) and *p*-values are shown. **c** ROC curves from predictive models of the presence of MMSE change in CI participants. The reference model (blue) and the reference model with p-tau181 levels (red) are shown. The 0.5 AUC reference line is shown in green. ROC curves for CN participants for either models are not shown. **d** AUC of ROC curves obtained by the reference model (Reference) and after adding plasma NfL (+NFL) or plasma p-tau181 levels (+PTAU) in both the CN and CI groups. *, *p*-value < 0.05

In CN participants, only NfL plasma levels were associated with changes in CDRSoB, while in the CI group NfL levels were associated with changes in CDRSoB, and p-tau181 levels were associated with changes in both CDRSoB and MMSE (Table 3). After controlling for age, sex, years of education, *APOE* ε4 status, baseline score, and time to follow-up; the association of plasma NfL levels with CDRSoB changes remained significant in CN participants, as well as the association of p-tau181 with MMSE change in CI participants (Fig. 3a). Applying backwards selection to determine the best predictive models identified plasma NfL as an independent predictor of changes in CDRSoB in CN participants and plasma p-tau181 as an independent predictor of MMSE changes in CI participants (Fig. 3b). Adding plasma p-tau181 levels to a reference model improved the prediction of a decline in global cognition in CI participants (Fig. 3c, d, *p*-value = 0.0318; sensitivity: 0.88; specificity: 0.69, Delong’s ΔAUC = 0.051). Despite their association with CDRSoB changes, plasma NfL levels did not contribute to improve prediction of disease severity progression when compared with a reference model (data not shown). Combinations of both plasma markers did not improve this prediction either (data not shown).

**Table 3:** Associations of plasma NfL and p-tau181 levels with CDRSoB and MMSE change

	ΔCDRSoB			ΔMMSE		
	Variable	Coeff.	*p*-value	Variable	Coeff.	*p*-value
CN	NfL	0.386	**0.001**	NfL	− 0.142	0.240
	p-tau181	− 0.056	0.620	p-tau181	0.030	0.801
CI	NfL	0.227	**0.035**	NfL	− 0.145	0.230
	p-tau181	0.252	**0.020**	p-tau181	**− 0.293**	**0.017**

Linear regression models with changes in CDRSoB (ΔCDRSoB) or MMSE (ΔMMSE) at last follow-up as dependent variables with plasma NfL or p-tau181 levels. For each variable, standardized coefficients (Coeff.) and significance are shown

## Discussion

We have found increased plasma NfL levels in CN participants with AD pathology, as compared with CN participants without AD pathology. Regression analysis found no associations between plasma NfL levels and AD pathology, however. Plasma p-tau181 levels were higher in the CI patients with AD and improved a model for AD prediction in CI participants. Further, plasma NfL levels were associated with clinical disease progression in the CN group while plasma p-tau181 levels were associated with decline in global cognition in the CI group. However, only plasma p-tau181 levels improved a reference model to predict cognitive decline in CI participants. Together, these results support the idea of plasma NfL levels as being a marker of neurodegeneration, but not specific for AD, and suggest plasma p-tau181 levels could be used for both the diagnosis of AD and the prediction of disease progression in memory clinic patients.

Plasma NfL levels were higher in CN with AD, CI non-AD, and CI with AD groups, when compared to CN non-AD participants. Previous studies have reported higher plasma NfL levels to be associated with AD dementia [3–6]. These studies defined AD using clinical assessment only [23] and did not consider CSF biomarkers for AD diagnosis. Accordingly, patients presenting clinically as AD dementia, but having cognitive impairment due to other cerebral pathologies may have been misdiagnosed and included, whereas our approach considers cerebral pathology as measured by CSF biomarkers. Additionally, we considered in our reference model known diagnosis covariates, including *APOE* ε4 status. We found that plasma NfL levels were not associated with the presence of AD pathology in either the CN or CI group. These results along with our finding of correlations between NfL and CSF tau, but not CSF Aβ1–42 and CSF Aβ42/Aβ40 ratio reinforce the role of NfL as a marker for neuronal injury [2], although not in an AD specific fashion as, in line with previous work, NfL appears independent of amyloid pathology [24].

Previous studies found associations of elevated plasma p-tau181 with amyloid positivity in participants with normal cognition or with cognitive impairment [8, 11, 12]. A previous study found that combining plasma p-tau181 levels with either CSF tau or p-tau181 increases the predictive performance of clinically defined AD [25]. Another study that defined AD considering both amyloid pathology and tau pathology reported results in line to ours, i.e., elevated p-tau181 levels in AD and predictive power in MCI and dementia participants [10]. The addition of plasma p-tau181 levels to a reference model including age, sex, years of education, and *APOE* ε4 status significantly improved the prediction performance for AD in CI patients. We observed a significant contribution of plasma p-tau181 independently of *APOE* ε4 in this model. Previous studies investigating the association of plasma p-tau181 with AD either did not consider the *APOE* genotype [12, 13], or they did not consider the effects of this factor independently of p-tau181 [9, 10]. Considering the *APOE* ε4 genotype as a covariate, we found that plasma p-tau181 has an independent and significant contribution to the prediction of AD in patients with cognitive impairment. This finding indicates that the combination of plasma p-tau181 and *APOE* ε4 genotype with clinical variables is superior to considering *APOE* ε4 genotype and clinical variables only to diagnose AD in memory clinic patients with cognitive impairment.

Together, our results indicate that plasma NfL levels can be used to identify participants with normal cognition at increased risk of having cerebral AD pathology and contributes to identifying neurodegeneration irrespective of the underlying cause. According to our findings, plasma NfL does not contribute to improving differential diagnosis of AD in memory clinic patients with cognitive impairment. On the other hand, plasma p-tau181 levels have an independent and significant contribution to the prediction of the presence of cerebral AD pathology and appear to be more specific than plasma NfL levels for AD pathology. Therefore, plasma p-tau181 may be more appropriate for differential diagnosis in memory clinic patients presenting with cognitive impairment. Other studies using different quantification methods [9, 26, 27] have also reported this association between plasma p-tau181 levels and AD, further supporting its usage as a blood-based biomarker of AD. Importantly, neither plasma biomarker was correlated to QAlb levels, indicating their levels are independent of blood-CSF barrier permeability.

The non-specificity of the association of plasma NfL with AD is further shown by correlations of plasma NfL with CSF tau levels, independently of cognitive status, while only a weak correlation with CSF p-tau181 in CN, and no correlation with CSF markers other than CSF tau were present in CI. In a previous study, plasma NfL was not associated with any CSF biomarker in CN participants and AD dementia patients, but in MCI participants it was associated with CSF Aβ1–42 and CSF tau [3]. Plasma NfL levels have been previously correlated to amyloid load assessed by PET scan in cognitively normal participants [28]. This suggests AD pathology might be the main cause of neuronal injury and therefore NfL increase in CN participants, while in a majority of patients in the CI group neuronal injury might be caused by other pathologies, rendering NfL inefficient for differential diagnosis in this later group. Conversely, plasma p-tau181 levels correlated with CSF p-tau181 in CN participants and with all CSF biomarkers in CI participants, reinforcing its role as a biomarker candidate useful for differential diagnosis of AD.

In both CN and CI groups, higher plasma NfL baseline levels were associated with more rapid increase in disease severity as indicated by CDRSoB change at follow-up. After controlling for possible confounders only the association of plasma NfL levels with CDRSoB changes in CN participants remained significant. Previous studies have reported plasma NfL levels to correlate with baseline cognition [3–7, 29–31]. Of these studies, only two considered both CDR and MMSE scores [4, 7], and a single study reported a correlation of plasma NfL levels with longitudinal MMSE change in cognitively impaired participants [3]. In line with previous reports [4–6], in CI patients higher NfL was associated with more marked increase in clinical disease severity over time. When added to a reference model based on clinical variables and the *APOE* ε4 status, plasma NfL did not significantly improve the prediction of severity progression at follow-up visit, however. Since NfL can be associated with neuronal injury of multiple aetiologies rather than with a specific pathological mechanism, elevated levels are indicative of multiple potential outcomes, rendering it inappropriate for modeling. Overall, our results suggest plasma NfL may be useful as a blood-based marker to identify individuals at high risk of cognitive decline among cognitively normal individuals.

We found higher plasma p-tau181 levels to be associated with more rapid increase in disease severity as well as with more marked decline in global cognition as assessed by changes in MMSE. Adding plasma p-tau181 levels to a reference model including age, sex, years of education, *APOE* ε4 status, baseline MMSE, and time to follow-up, significantly improved the prediction of decline in global cognition in CI participants. While the association of high levels of plasma p-tau181 with cognitive decline has been previously observed in MCI patients [13], we show here the added value of this plasma marker to predict cognitive decline when combined with other non-invasive measures. While these findings remain to be confirmed in an independent cohort, they suggest the utility of plasma p-tau181 in clinical practice as a blood-based prognostic biomarker for cognitive decline, in particular in patients with cognitive impairment.

### Limitations

A limitation of this study is the relatively small number of included participants with dementia, preventing us to specifically address the performance of the plasma biomarker candidates in this subgroup. Our work does however benefit from the inclusion of both elderly participants with normal cognition and memory clinic patients with cognitive impairment, allowing the assessment of differential diagnosis utility. Furthermore, we used established CSF biomarkers of cerebral AD pathology to define AD at both the asymptomatic and the clinical stage, enabling to address relationships to cerebral pathology while ensuring cognitive impairment due to other cerebral pathologies was not misdiagnosed as AD. Additionally, we have considered multiple covariates in this study, therefore assessing the specific clinical relevance of plasma NfL and p-tau181 levels.

## Conclusions

We have investigated the associations of plasma NfL and p-tau181 levels with CSF biomarkers of amyloid, neuronal injury, and tau pathology, and the predictive performance of the plasma marker candidates for cerebral AD pathology and cognitive decline. Our results suggest that plasma NfL may be useful as a blood-based marker to identify cognitively normal individuals at risk of cognitive decline. Plasma p-tau181 levels can serve as a predictive blood-based biomarker of both AD pathology and cognitive decline, but its performances depend on whether it is used in cognitively normal older individuals or in patients with cognitive impairment. While these findings need further validation in independent samples before use in clinical practice, they show the potential utility of blood-based biomarkers in both older individuals with normal cognition and memory clinic patients with cognitive impairment.

## Data Availability

The datasets used and/or analyzed during the current study are available from the corresponding author on reasonable request.

## Abbreviations

Aβ_1–42_: CSF β-amyloid 1–42 peptide
AD: Alzheimer’s disease
APOE: Apolipoprotein E
AUC: Area under the curve
CDR: Clinical dementia rating
CDRSoB: CDR sum of boxes
CSF: Cerebrospinal fluid
CN: Normal cognition group
CI: Cognitive impairment group
MCI: Mild cognitive impairment
MMSE: Mini-Mental State
NfL: Neurofilament light
p-tau181: Tau phosphorylated at threonine 181
QAlb: Albumin CSF/serum quotient
tau: Total-tau
VIF: Variance inflation factor
